# Application of 16S rRNA virtual RFLP for the discrimination of some closely taxonomic-related lactobacilli species

**DOI:** 10.1186/s43141-022-00448-8

**Published:** 2022-12-16

**Authors:** Nora Laref, Khadidja Belkheir

**Affiliations:** University Ahmed Zabana of Relizane, Relizane, Algeria

**Keywords:** Virtual RFLP, Restriction enzymes, *Lactobacilli*

## Abstract

**Background:**

Several species in *Lactobacillaceae* family were recognized as potential probiotic bacteria. In this group of lactic acid bacteria, species are taxonomically closed and usually share similar 16S rRNA gene, thus, instead of so their identification and discrimination are too difficult.

**Method:**

In the present study, virtual restriction fragment length polymorphism (RFLP) is instead of was used as a tool to discriminate between the closely related species *Lactiplantibacillus plantarum* (*L plantarum*), *Lactiplantibacillus paraplantarum* (*L paraplantarum*), and *Lactiplantibacillus pentosus* (*L pentosus*); *Latilactobacillus sakei* (*L sakei*), *Latilactobacillus curvatus*(*L curvatus*), and *Latilactobacillus graminis* (*L graminis*); *Lacticaseibacillus casei* (*L casei*), *Lacticaseibacillus paracasei* (*L paracasei*), *Lacticaseibacillus zeae,* and *Lacticaseibacillus rhamnosus*; *Lactobacillus gasseri* (*L gasseri*) and *Lactobacillus johnsonii* (*L johnsonii*). In silico comparative analysis of 16S rRNA sequences digested by 280 restriction enzymes was performed in order to search the key enzymes which gives different profiles.

**Results:**

Results revealed that *L casei*, *L paracasei*, *L zeae*, and *Lb rhamnosus* could be separated from each other on the basis of AlwI, BpuEI, BsgI, BsrDI, BstYI, EarI, MluCI, and NsPI RFLP. Results showed also that different RFLP patterns were obtained from *L sakei*, *L graminis* and *L curvatus* by using both AflI and NspI endonucleases (in separated restriction) and *L plantarum*, *L paraplantarum*, and *L pentosus* were distinguished each one from the other by MucI, NspI, and TspDTI PCR-RFLP. *Lb gasseri* and *L johnsonii* were also separated on the basis of Mse I, Taq I, and Dra I RFLP.

**Conclusion:**

In this study, we proved that too closely related species could be separated in virtual analysis on basis of their 16S rRNA RFLP patterns using key restriction enzymes method.

## Background

Lactobacilli is the largest and more diverse group of lactic acid bacteria. It consists of a high number of species isolated from several ecological niches and reported as potential industrial and probiotic bacteria for most of them [1]. For the extreme diversity of lactobacilli species, their classification has been constantly reshuffled. At first, these species were divided into 3 groups on the basis of their phenotypic carbohydrate fermentation and optimal temperature growth [2, 3]. However, the phenotypic typing methods are not completely accurate, and it was difficult to associate the phylogeny of some lactobacilli species showing intermediate characteristics with their phenotypes [3–5]. Therefore, using newer molecular taxonomic methods based on genome analysis has become common among researchers with the aim to improve the classification of lactobacilli species. But methods based on genome analysis have been usually reported as time-consuming, expensive, and not always reliable [6–8]. On the basis of 16S rRNA gene analysis, *Lactobacillus* genus was first of all divided into 7 or 8 groups [9]. Then, Salvetti et al. [2] updated the classification of this genus into 15 groups of three or more species by phylogenetic analysis of 16S rRNA gene sequence. Six years later, this genus was reclassified into 18 groups using the analysis of 16S rARN phylogeny, analysis of the whole genome sequence and the analysis of amino acids percentage identity in conserved proteins [10]. In another polyphasic approach based on the analysis of overall genome-relatedness indices and metabolic or ecological properties of the organism, the taxonomic relationship between *Lactobacillus* species was recently re-evaluated. Today, lactobacilli group is divided into 25 genera including *Lactobacillus delbrueckii* group, *Paralactobacillus* and 23 novel genera with new nomenclature classification [1]. But the addition of new species each year will require powerful tools offering high throughput, reliable and rapid analysis. The use of nucleic acids sequences already available on nucleotide database NCBI and bioinformatics tools provide the opportunities to analyze rapidly more information of microbial species [8, 11].

The aim of this study was to develop an easy and fast method to accurately distinguish between too reliable closely species in lactobacilli group by analysing in silico at the same time many restriction digest profiles of 16S rRNA with a lot of enzymes, to search the key enzymes which give different profiles. Such approach could also give solutions to students and researchers working on lactobacilli in laboratories with limited academic resources.

## Method

We download sequences of partial 16S rRNA gene of *L gasseri*, *L johnsonii*, *L casei*, *L paracasei*, *L rhamonosus*, *L zeae*, *L plantarum*, *L paraplantarum*, *L pentosus*, *L curvatus*, *L graminis*, and *L sackei* closed species listed in http://www.bacterio.net/lactobacillus.html from

GenBank (http://ncbi.nlm.nih.gov). All sequences were aligned with MAFFT program (https://mafft.cbrc.jp/alignment/server) [12]. Sequences were then subjected to a virtual restriction mapping with the pDRAW32 software to find the restriction key enzymes. In the first stage we selected enzymes which cut in maximum *n* − 1 and in minimum one sequence where *n* is the number of sequences aligned, then in the second stage, endonucleases which cut just one sequence were considered as the key enzymes.

Only closely related species showing high degree (more than 99%) of 16S rRNA gene sequences similarity and are difficult to be separated are used in this study and listed in Table 1.

**Table 1:** Accession number and length of partial 16S rRNA of some closed lactobacilli species

	Accession number	Length of partial 16S rRNA (bp)
*L paracasei*	AB289225	670
*L casei*	EF468100	619
*L zea*	AY196979	506
*L rhamnosus*	MW040507	518
*L curvatus*	AB289077	673
*L sakei*	AF429523	512
*L graminis*	AB289145	651
*L plantarum*	EF468099	561
*L paraplantarum*	AB289239	626
*L pentosus*	AB289240	622
*L gasseri*	AY341531	583
*L johnsonii*	KF267449	579

## Results

The in silico prediction of the restriction patterns of partial 16S rRNA (sequences length ranging from 506 to 673 bp) after alignment of some related closed species belonging to *Lactobacillus* genus and L plantarum*-*, L casei*-*, L curvatus groups were made by restriction enzymes.

**Fig. 1:**
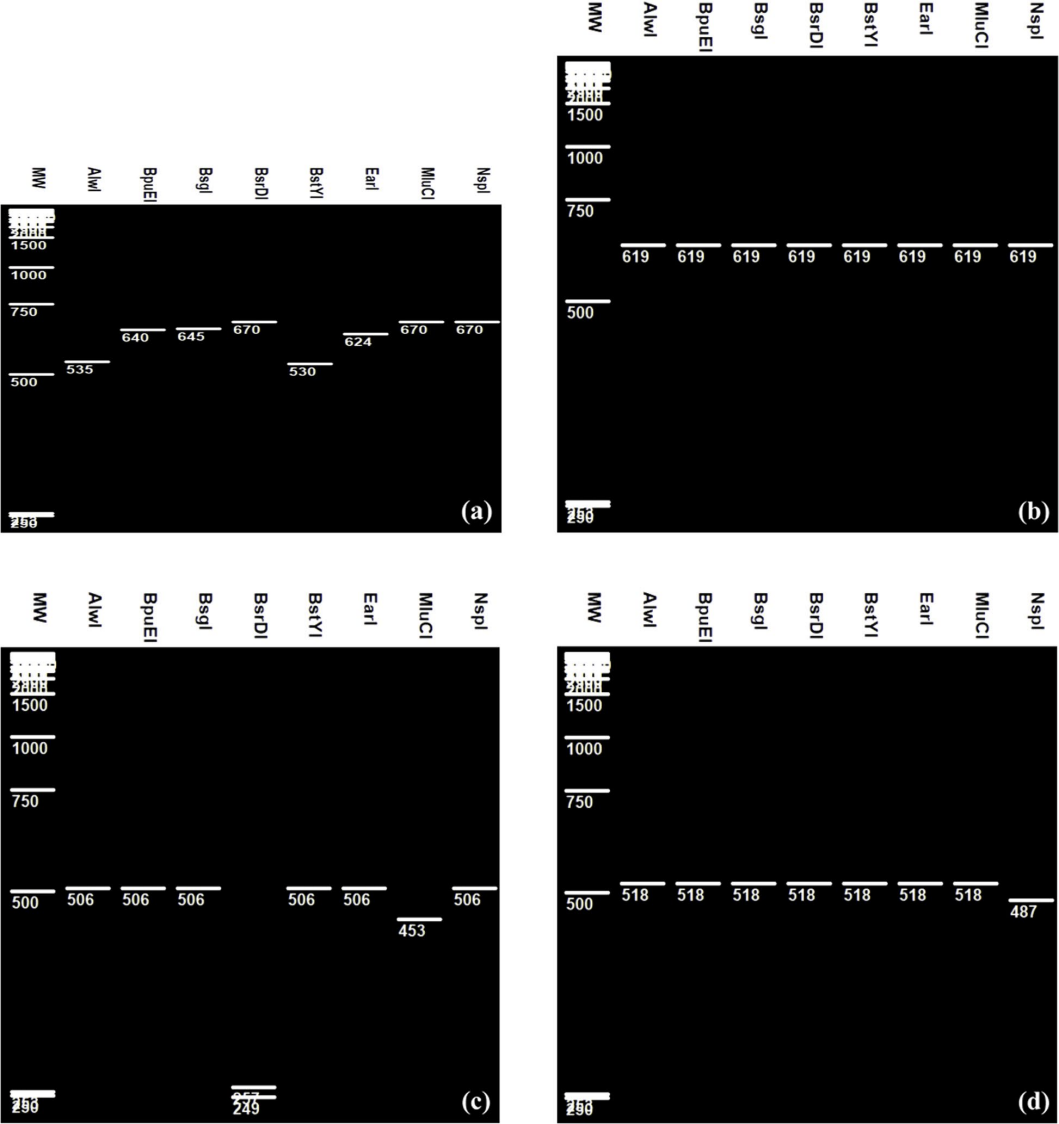
Virtual RFLP of **a ***L paracasei* and **b ***L casei*, **c ***L zea*, **d ***L rhamnosus*

Restriction fragment length polymorphysm (RFLP) of sequences of approximately 670, 619, 518, and 506 bp consistent with the partial 16S rRNA genes obtained from *L paracasei*, *L casei*, *L rhamonosus*, and *L zeae* respectively indicated different banding patterns after digestion by AlwI, BpuEI, BsgI, BsrDI, BstYI, EarI, MluCI, and NsPI (Fig. 1). *L casei* could be easily separated from the three others closed species in this group because no restriction was indicated for all these enzymes (Fig. 1b). However, unique restriction site were shown by AlwI, BpuEI, BsgI, BstYI, and EarI on the *L* paracasei gene and by NspI on *L rhamnosus* gene and by MIuCI on *L zeae* gene (Fig. 1). RFLP patterns of *L paracasei* yielded fragments ranging from 530-to 645 bp and resulted in a well separated band for each one (Fig. 1a). Fragments of approximately 487 and 453 bp were also observed for *L rhamonous* and *L zeae* respectively when using NspI and MIuCI endonucleases (Fig. 1c, d). On the other hand *L zeae* gene could be also digested by BsrDI endonuclease which yields in two fragments of 257 and 249 bp (Fig. 1c). These results indicated rapid discrimination of these four closely related species within the *L casei* genus by using such key endonucleases.

**Fig. 2:**
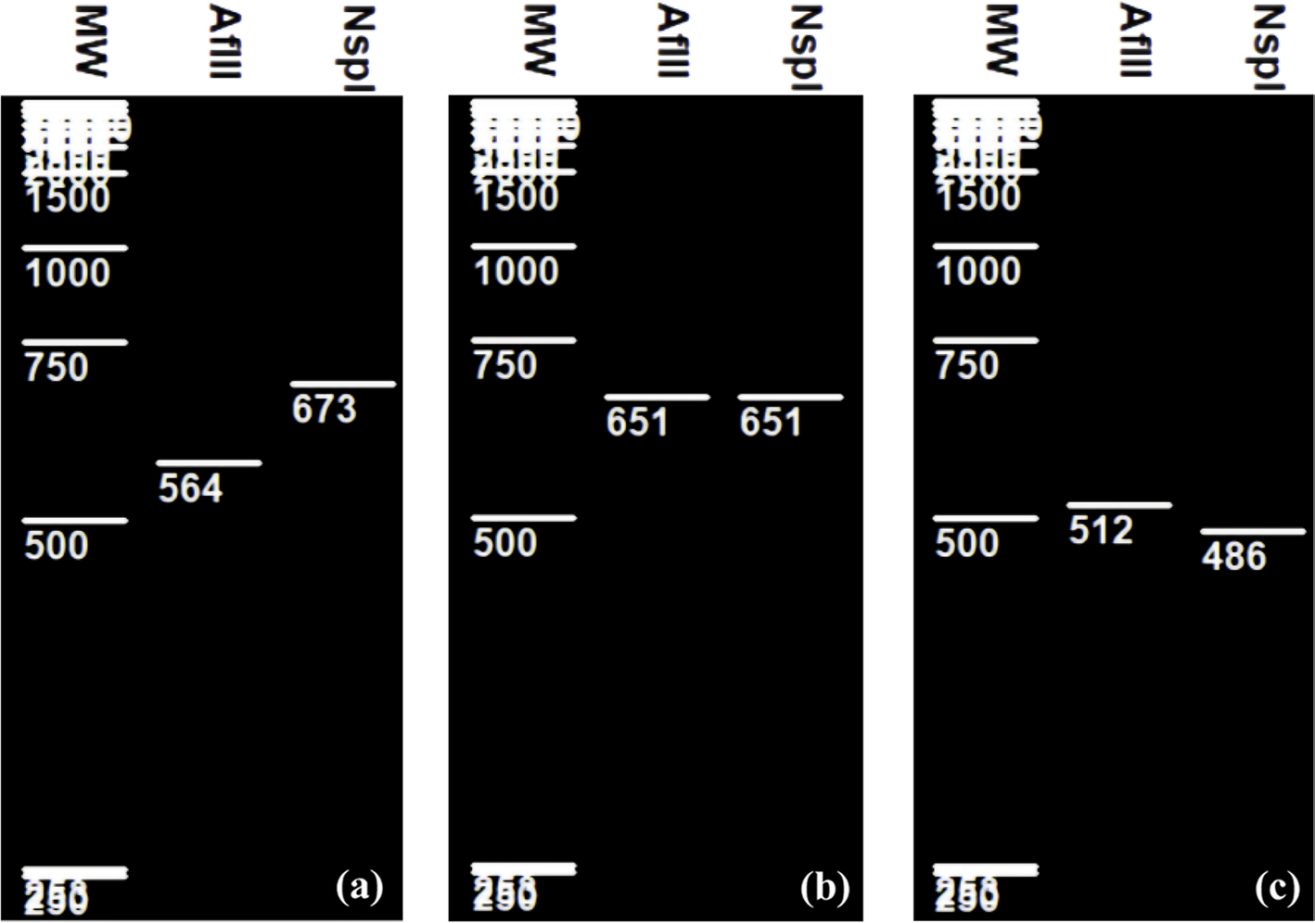
Virtual RFLP of **a ***L curvatus*, **b ***L graminis*and, **c ***L sakei*

The RFLP patterns obtained by using both endonucleases AflI and NspI (in separate restrictions) on 16S rRNA genes fragments of *L curvatus* (673 bp), *L sakei* (512 bp), and *L graminis* (651 bp) can allow differentiation of these 3 species (Fig. 2). Effectively AflI restriction patterns showed a band of approximately 564 bp for *L curvatus*, while no digestion was noted for the two remaining species (Fig. 2a–c) and at least one NspI restriction site exists in the 16S rRNA gene of the *L sakei* which exhibited one fragment of 486 bp after digestion by this enzyme as shown in Fig. 2c.

**Fig. 3:**
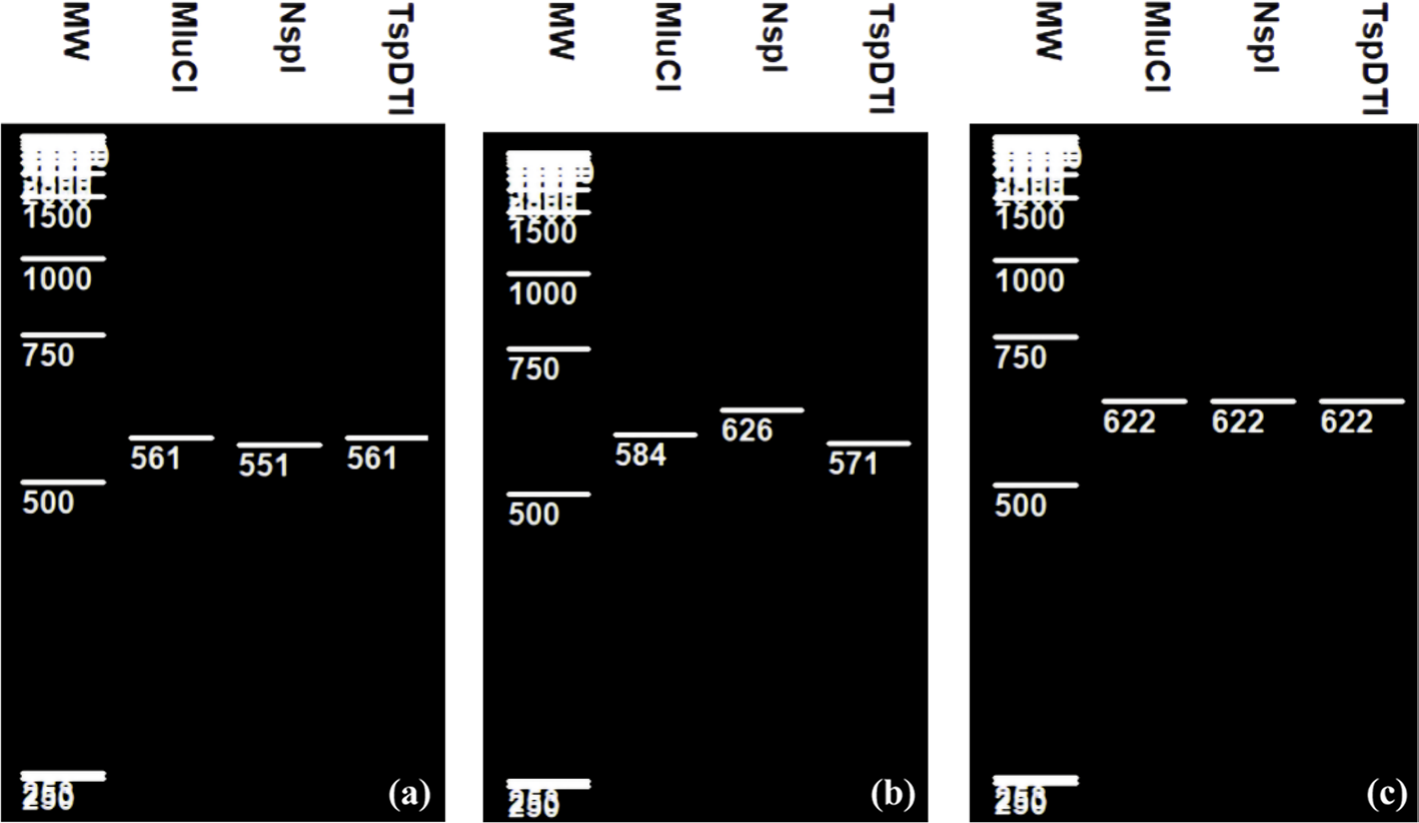
Virtual RFLP of **a ***Lplantarum*, **b ***Lparaplantarum* and **c ***L pentosus*

The analysis of the MucI, the NspI, and the TspDTI PCR-RFLP in silico patterns of the 561 bp, 622 bp, and 626 bp corresponding to the partial 16S rRNA genes fragments of *L planatarum*, *L pentosus*, and *L paraplantarum* respectively indicated that these three closely related species were clearly differentiated (Fig. 3). *L plantarum* could be rapidly discriminated from *L paraplantarum* species by using NspI which produced distinct restriction patterns from these two species, it cleaved and generated one fragment of approximately 551 bp in the first one and not digested in the second one (Fig. 3). Analysis with MucI or TspDTI restriction enzymes produced also different restriction profiles from these two species. They showed a single recognition site for each of them in *L paraplantarum* and generated two bands, 584 bp and 571 bp respectively for MucI and TspDTI but no digestion was observed for both enzymes in *L plantarum* (Fig. 3a). Results also showed no digestion PCR products from *L pentosus* when using the three considered endonucleases (Fig. 3c).

**Fig. 4:**
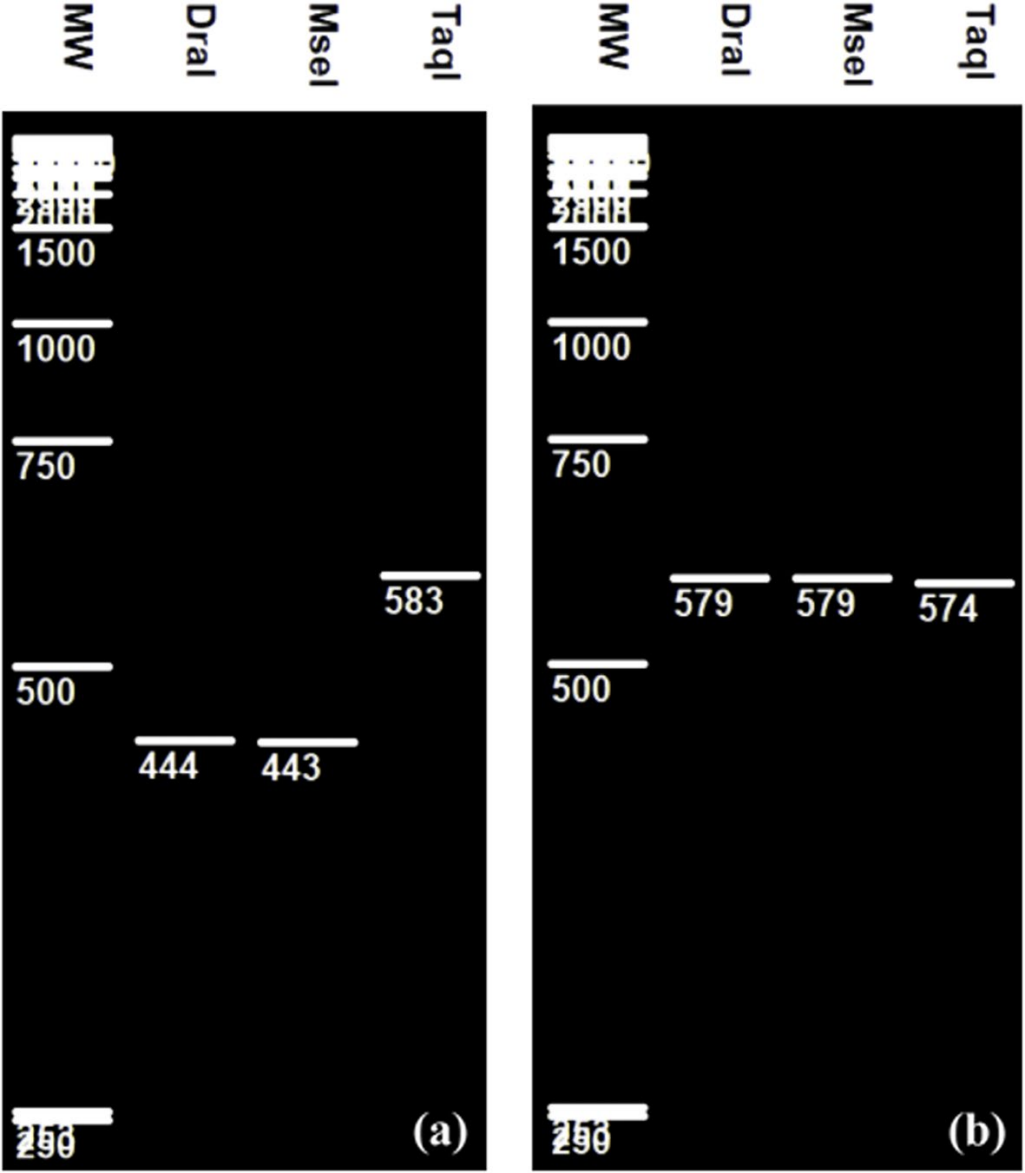
Virtual RFLP of **a ***L gasseri* and **b ***L johnsonii*

Others RFLP patterns corresponding to the closely related *L gasseri* and *L johnsonii* partial 16S rRNA digestion by DraI, MseI and TaqI showed unique restriction site for these three key enzymes (Fig. 4). Fragments of 443 and 444 bp were obtained after the digestion of *L gasseri* partial gene (583 bp) by MseI and DraI respectively (Fig. 4a, b) and one fragment of about 574 bp resulted from the digestion of *L johnsonii* partial gene (579 bp) by TaqI (Fig. 4b).

## Discussion

*Lactobacilli* is the largest and most heterogeneous group among lactic producing bacteria. It is composed of several species commonly used as starter cultures and probiotics. Due to their economical interest, the precise identification of species in this group often requires molecular identification [8]. The taxonomy of *lactobacilli* became clearer after the genome sequencing technologies appearance and *L plantarum* WCFS1 genome was the first to be sequenced [13]. 16S rRNA sequences were widely used for the first diagnostics and classification of bacterial species because extensive databases of sequences, primer sets, and enzymes for analysis of 16S rRNA length polymorphism are well established [14, 15]. However, some species within *lactobacilli* groups share similar 16S rRNA genes (more than 99%) and are undistinguishable on basis of their 16S rRNA phylogeny [16, 17]. The use of RFLP of 16S rRNA genes resulted in efficient discrimination of lactobacilli except for some species in L casei-, L plantarum- groups and *Lactobacillus* genus for which limitations were encountered specially to separate *L casei* from *L paracasei* and *L plantarum* from *L paraplantarum* [17–20]. Likewise, there were some difficulties in distinguishing *L zeae* from *L rhamonosus* and *L casei* or *Lb gasseri* from *Lb johnsonii* on basis of 16S rRNA phylogenetic [11, 14, 21]. For all these authors the correct choice of restriction endonucleases was suspected. Therefore, other molecular approaches like SDS-PAGE protein profiles [22], PFGE fingerprinting [18], protein-encoding genes as *hsp60* marker [14, 23], and *dnaK* marker [16, 17] have been added to the RFLP analysis for better discrimination of lactobacilli closed species. Approaches based on analysis of *recA* gene, partial *Tuf* gene, *mal* gene, *pepC* gene, *pepN* gene, *htrA* gene, and *rpoB* gene were also used in cases of species sharing more than 99% 16S rRNA sequences similarities [21, 24].

As pointed out in Figs. 1, 2, 3, and 4 different RFLP patterns were obtained by the selected restriction endonucleases making it possible to distinguish clearly between *L casei*, *L paracasei*, *L zeae*, and *L rhamnosus*; between *L plantarum*, *L paraplantarum*, and *L pentosus*; between *L gasseri* and *L johnsonii*; and between *L curvatus*, *L sakei*, and *L graminis*.

*L casei* and *L paracasei* were usually confused each one to the other because of the closed relationship between many strains of *L paracasei* species and the *L casei* type strain ATCC 393 [20]. Results illustrated in Fig. 1 showed that *L casei* and *L paracasei* could be discriminated effectively on the basis of their RFLP patterns by using AlwI, BpuEI, BsgI, BsrDI, BstYI, EarI, MluCI, and NsPI among the restriction endonucleases tested in silico. It is interesting to note that real digestion by restriction enzymes resulted usually in similar fragment sizes to those in the in silico experiments [25]. However, the use of the inadequate enzymes limited some authors to distinguish *L rhamnosus* and *L paracasei* from the *L casei* type strain on basis of small-fragments (PCR product of approximately 295 bp) by Not1 restriction enzyme patterns and neither by using large-fragment PFGE [18] or on basis of PCR amplification and digestion products (fragments of 1500 bp in size) using AluI and MspI restriction enzymes [20]. There has been also a controversy about the classification of *Lb zeae* which was usually classified as a subspecies of *Lb casei* or *Lb rhamnosus* [21]. These species were differentiated from its closest neighbour only when considering other markers like Dnak-PCR RFLP/apoI [16], Tuf-PCR RFLP/HaeIII [21], or 16S-23S rDNA ITS-PCR RFLP/MseI [26]. Our results indicate that the partial 16SrRNA RFLP using both BsrDI and MluCI key enzymes are valuable method to differentiate *Lb zeae* although 16S rRNA gene is significantly less polymorphic than other genes because similarities are significantly higher in 16S rRNA sequences (from 98.9 to 99.9%).

Also, the discrimination between *L plantarum*, *L pentosus*, and *L paraplantarum* species produced ambiguous outcomes because molecular analysis of 16S rRNA polymorphism by some endonucleases was not sufficient enough to reveal significant differences [16, 18, 20]. Huang and Lee [16] noted also that species in L plantarum group were indistinguishable using HaeIII, MspI, and AluI for dnaK amplicons digestion. These two authors pointed that the crucial element in RFLP techniques is the good selection of the restriction enzymes [16]. In addition, hsp60 RFLP patterns obtainable by using both endonucleases AluI and TacI were insufficient to distinguish between *L plantarum* and *L pentosus* [23]. In our study, we showed that MucI, NspI, and TspDTI selected as key enzymes produced three different restriction profiles and distinguished these three related species.

On the other hand, comparison of AflI and NspI restriction enzyme patterns showed good species distinction between these following species of *L curvatus*, *L sakei* and *L graminis.* Similar to our finding (data not shown) previous in vitro restriction analysis using Hind III endonuclease discriminates *L sakei* from *L curvatus* but no data were reported for *L graminis* species [27]. In the present study, only restriction enzyme showing at least one sequence digestion are selected, therefore Hind III endonuclease could not be considered as key enzyme because it showed digestion in both *L curvatus* and *L graminis* (data not shown).

In the case of *L gasseri* and *L johnsonii* belonging to *Lactobacillus* genus, 16S rRNA gene sequence analysis is not able to reveal significant differences between these two species and their differentiation leads usually to ambiguous results using several powerful approaches like MALDI-TOF MS [26], for this reason, various molecular tools have been combined for the precise differentiation of *L johnsonii* from *L gasseri* [14, 28, 29].

In the present study, we showed that the partial 16S rRNA RFLP generated by the key enzymes DraI, MseI, and TaqI could rapidly differentiate between *L gasseri* and *L johnsonii* although their highest sequences homologies [11]. A previous study showed that these two closed species could also differentiate each one from the other on basis of ITS 16S-23S rDNA RFLP/TaqI but not with ITS RFLP/MseI [26].

## Conclusion

Results of this study confirmed that in silico using key enzymes could differentiate between some closely related lactobacilli at species level. This approach could be used as an initial step for rapid and reliable classification of some lactobacilli closed species. Nonetheless, one major limitation was encountered when conducting the present study. It is clearly shown that the number of analysed sequences must be reduced to avoid confusion in selecting the key enzymes. For this reason, authors are currently working on a new technique to resolve this limit.

## Data Availability

Not applicable.

## Abbreviations

NCBI: National Center for Biotechnology Information
PCR: Polymerase Chain Reaction
PFGE: Pulsed-field Gel Electrophoresis
RFLP: Restriction Fragment Length Polymorphism
SDS-PAGE: Sodium Dodecyl Polyacrylamide Gel Electrophoresis
